# Flowers, Ferns, and Ward Decorations

**Published:** 1886-11-27

**Authors:** 


### FLOWERS, FERNS, AND WARD DECORATIONS.

[Continued from page 111.—Communications for this column should be addressed "Gardener," care of the Editor of THE HOSPITAL.]

**Flower Services.**

It is more than disappointing to nurses and patients alike when a hamper (through whose crevices little bits of green betoken the presence of flowers) is unpacked, to discover nothing but a tumbled mass of leaves and petals, mashed into a gelatinous muddle,—"fit for nothing but the dust-bin," as I once heard a nurse remark, who was thus suffering from the after-results of a rural "flower service," packed in the usual happy-go-lucky way, and presenting the usual forlorn heap on the floor of her ward. All this because people will not pack properly. There is much to be said in favour of Flower Services, much to fill the orthodox mind with respect and admiration, and much to deplore. People who undertake to benefit their neighbours in this manner would do better if their common sense equalled their benevolence. They need not to be "too thoughtful, but just thoughtful enough" in the packing department, the rock ahead whereon the unwary founder so often splits.

**Worse than Useless.**

The sight of hundreds of little children marching up the church aisles, in a well-ordered, gaily coloured procession, to lay their offerings of bloom at the foot of God's altar-table is all very nice and very pretty. But when these bouquets have been, as in many cases gathered overnight, and forwarded by train, to be subsequently held tightly in hot little nervous hands for hours at a stretch, then if taken direct to the hospital, they would be worn out and useless. Unfortunately the hot little hands are not the last or the worst trial of such flowers. The children deposit their offerings in a heap, and then the flowers are tossed into baskets to ferment sometimes for hours before their removal to the hospitals. It will surprise no one to hear that when they ultimately get there, little remains to be done by the hapless individuals in charge but to get rid as soon as possible of a pulpy, floricultured salad, and to return the hampers which contained it to their respective proprietors. Such an end to the floral offerings of a very useful service ought to be prevented by the exercise of a little necessary care and good management on the part of those responsible.

**How to Pack Flowers.**

In cases, however, where the proceeds of such services can be brought direct to their destination, it follows naturally enough that the charitable object does not fall short of its appointed end. Everyone knows how the inmates of hospitals and workhouses welcome the gifts of flowers, brought straight from church immediately after the service is concluded, for then they are fresh and sweet, and still possessed of their own original shapes and colours. Even, however, when this cannot be done, and a railway journey is inevitable, things might turn out better if due attention were only bestowed on the packing. This should be undertaken with a view to prevent the blossoms shaking about on the way, the unavoidable vibration of the train being quite as much as is good for them in that respect. If they are tied up in reasonable-sized bunches, and placed close together in the baskets, with no room to get loose and tumble about, there is little fear of the dust-bin being called into requisition at the journey's end. In all cases but the most fragile, the flowers should be kept as dry as possible; when they have been kept soaking in water for any length of time before being sent away, they fail to present a very creditable appearance on their arrival. They should be gathered the last thing before packing, as late in the day as can be, just in time to catch the last evening train, to travel through the night and avoid the heat of day time; and, above all, let them go by "passenger trains." The cost is greater, but so is the security; and I understand that most railway companies will take things of the sort for half-price, when informed they are for the use of the sick poor.

